# Plasma pepsinogens, antibodies against *Helicobacter pylori*, and risk of gastric cancer in the Shanghai Women's Health Study Cohort

**DOI:** 10.1038/bjc.2011.77

**Published:** 2011-03-15

**Authors:** C C Abnet, W Zheng, W Ye, F Kamangar, B-T Ji, C Persson, G Yang, H-L Li, N Rothman, X-O Shu, Y-T Gao, W-H Chow

**Affiliations:** 1 Division of Cancer Epidemiology and Genetics, National Cancer Institute, Executive Plaza South, Room 320, 6120 Executive Boulevard, MSC 7232, Rockville, MD 20852, USA; 2 Division of Epidemiology, Department of Medicine, Vanderbilt Epidemiology Center, Vanderbilt-Ingram Cancer Center, Vanderbilt University School of Medicine, Nashville, TN, USA; 3 Center for Health Services Research, Department of Medicine, Vanderbilt University, Nashville, TN, USA; 4 Department of Medical Epidemiology and Biostatistics, Karolinska Institutet, Stockholm, Sweden; 5 Department of Public Health Analysis, School of Community Health and Policy, Morgan State University, Baltimore, MD, USA; 6 Department of Epidemiology, Shanghai Cancer Institute, Shanghai, China

**Keywords:** gastric cancer, pepsinogens, *Helicobacter pylori*, cohort, China

## Abstract

**Background:**

Circulating pepsinogens can indicate atrophic gastritis, a precursor of gastric cancer. We tested the association between gastric cancer and plasma pepsinogens and antibodies against *Helicobacter pylori* in a case–control study nested in a prospective cohort.

**Methods:**

We selected 141 gastric cancer cases and 282 incidence-density sampled controls. Plasma concentrations of pepsinogens 1 and 2 were measured using ELISA kits, and anti-*H. pylori* antibodies were measured using a kit specific to Chinese strains. Associations were estimated using conditional logistic regression models adjusted for potential confounders.

**Results:**

Gastric cancer subjects were more likely to be anti-*H. pylori* positive than controls, 97 *vs* 92%. A plasma pepsinogen 1 (PG1) concentration <50 ng ml^–1^ (15% of cases) was associated with a significantly increased risk of gastric cancer (OR 4.23; (95% CI: 1.86–9.63), whereas a plasma pepsinogen 2 (PG2) concentration >6.6 ng ml^–1^ (75% of cases) was also associated with a significantly increased risk of gastric cancer (OR 3.62; (95% CI: 1.85–7.09). We also found that the PG1 : 2 ratio had a nearly linear association with gastric cancer risk.

**Conclusion:**

Lower plasma PG1 : 2 ratios are associated with a higher risk of gastric cancer. Furthermore, it appears that circulating pepsinogens 1 and 2 may be independently associated with the risk of gastric cancer.

Atrophic gastritis, a condition characterised by loss of glands and specialised cells in the stomach, is a precursor lesion of gastric cancer (Correa, 1992; Sipponen and Graham, 2007), the second most common cause of cancer death worldwide (Kamangar *et al*, 2006b). Atrophic gastritis may be diagnosed by histological examination of gastric biopsies, measuring gastric maximum acid output, or measuring serum or plasma concentration of proteins released from the gastric cells, such as pepsinogens (Samloff *et al*, 1982; Ley *et al*, 2001). Pepsinogens are proteinases that are mainly secreted by gastric cells and are classified into two major types: pepsinogen 1 (PG1) and pepsinogen 2 (PG2) (Samloff and Taggart, 1987). PG1 is secreted from the gastric fundic mucosa and PG2 is secreted from the cardiac, fundic, and antral mucosa of the stomach, and also from the duodenal mucosa (Samloff and Taggart, 1987). *Helicobacter pylori* infection is a strong risk factor for the development of both atrophic gastritis and gastric cancer (Forman *et al*, 1991; Parsonnet *et al*, 1991; Nomura *et al*, 2005; Kamangar *et al*, 2006a).

Few studies have examined the association between PG2 alone and gastric cancer risk, but a low concentration of PG1 or a low PG1 to PG2 ratio (PG1 : 2 ratio) in the serum or plasma is an indicator of atrophic gastritis and is associated with an elevated gastric cancer risk (Stemmermann *et al*, 1987; Fukuda *et al*, 1995; Ren *et al*, 2009). Most previous studies have used specific cut points to determine low levels of PG1 or PG1 : 2 ratio, but these cut points have varied across studies (Weck and Brenner, 2006; Brenner *et al*, 2007). Some of these cut points were initially determined by comparing the circulating PG1 : 2 ratio to a presumed reference standard, histological examination of gastric biopsies. However, histological examination of gastric biopsies cannot definitively diagnose atrophic gastritis because only a very small proportion of the stomach mucosa is examined, which may lead to sampling error (Satoh *et al*, 1998), and because of considerable interobserver variability in histological readings (Andrew *et al*, 1994). An alternative approach may be to examine PG concentrations as continuous or ordinal variables. Two recent studies have shown that using serum PG1 : 2 ratio as quartiles or as a continuous variable was more informative in predicting the risk of gastric cancer (Ren *et al*, 2009) or oesophageal squamous dysplasia (Kamangar *et al*, 2008).

We conducted this prospective nested case–control study to examine different methods of using PG1, PG2, or the PG1 : 2 ratio in assessing future risk of gastric cancer in a population-based cohort of women in Shanghai, China.

## Materials and methods

### Study participants

The case and control subjects were selected from women who participated in the Shanghai Women's Health Study (SWHS) and provided a blood sample. Details of the study design have been previously published (Zheng *et al*, 2005). In brief, a total of 81 170 women aged 40–70 years and residing in seven urban communities of Shanghai were invited to participate in the study. Of these, 75 221 (93%) women completed the baseline survey between 1996 and 2000. After exclusion of study subjects <40 or >70 years of age and those who had malignancies at baseline, 73 222 women remained in the analytic cohort. A total of 154 incident gastric cancer cases were diagnosed through December 2005, of whom 141 had plasma available for *H. pylori* and pepsinogen assays and were included as cases in this study. Two controls were matched to each case for menopausal status at sample collection, age (±2 years), date of sample collection (1 month), time of sample collection (morning or afternoon), and time interval since last meal (2 h). Controls were also free of any cancer at the time of cancer diagnosis for their corresponding case. No subjects were allowed to be sampled multiple times.

### Collection of data and biological samples

At study baseline, after obtaining informed consent, information on demographic characteristics, education and income, lifestyle and habits, diet, and several other factors were obtained via a combination of self-administered questionnaires and in-person interviews. Among cohort members, 56 831 (76%) women provided a 10 ml blood sample drawn into an ethylene diamine tetraacetic acid Vacutainer tube (Becton, Dickinson and Company, Franklin Lakes, NJ, USA). Samples were kept in a portable insulated bag with ice packs (0–4 °C) and processed within 6 h for long-term storage at −70 °C. Each woman also filled out a biospecimen collection form at the time they provided the sample.

### Outcome assessment

Outcome ascertainment was conducted by in-person interviews, and by annual record linkage to the population-based Shanghai Cancer Registry and the Shanghai Vital Statistics Unit. Participants were followed up by an in-home visit every 2 years to record details of their interim health history, including any cancer diagnosis and by record linkage with Shanghai Tumor Registry database. In the follow-up surveys, interviewers were able to interview and follow-up with 99.8% (2000–2002), 98.7% (2002–2004), and 96.7% (2004–2007) of cohort members or their next of kin. For cancer patients, information on date of diagnosis was collected and medical charts and diagnostic slides were reviewed to verify diagnosis.

### Pepsinogen assays

Plasma PG1 and PG2 were measured using an enzyme-linked immunosorbent assay (Biohit ELISA kit, Biohit, Helsinki, Finland) at the Karolinska Institutet, Sweden, by trained personnel unaware of subjects’ case status according to the manufacturer's instructions.

The quality control samples provided with the kits were included on each assay plate with additional quality control samples using pooled plasma from cohort members. The kit quality control samples showed CVs of 3.1 and 3.7% for PG1 and PG2, respectively. The cohort plasma quality control samples (two per plate and 14 in total) showed CVs of 5 and 17%, respectively.

### *H. pylori* assays

Plasma was evaluated for IgG antibodies to whole-cell and CagA *H. pylori* antigens using the China-specific ELISA (Biohit ELISA kit) and immunoblot (Helicoblot 2.0; Genelabs Diagnostics, Singapore) assays, respectively. All assays were done in Karolinska Institutet, Sweden. The kit quality control samples showed a CV of 6.4%. For the pooled plasma sample the CV was 4.5%. For immunoblot analysis, we used the criteria recommended by the manufacturer to interpret the CagA bands.

Similar to previous studies, *H. pylori* seropositivity cut point was defined as optical density ratios ⩾1.0 for the whole-cell antibodies (Kamangar *et al*, 2006a, 2007). *H. pylori* positivity was analysed as a dichotomous variable. Positive subjects were defined as those positive for whole-cell antibodies or CagA immunoblot, and negative subjects were those negative for both markers.

### Statistical analysis

Statistical analyses were done using SAS version 9.1 (SAS Institute Inc., Cary, NC, USA). We used two-sided *P*-values throughout the paper and considered those <0.05 to be statistically significant. We calculated Spearman's rank correlations between plasma concentrations of PG1 and PG2 and tested for differences in the distribution of concentrations using Wilcoxon tests.

In accord with the matched design of the case–control study, we used conditional logistic regression models to estimate adjusted odds ratios (ORs) and 95% confidence intervals (95% CIs) for the association between gastric cancer risk and plasma concentrations of PG1, PG2, or the PG1 : 2 ratio. There are no universally accepted cutoff points for dichotomising PG1 or the PG1 : 2 ratio (Weck and Brenner, 2006), and hence to be consistent with previous studies in China in which we used Biohit kits (Kamangar *et al*, 2008; Ren *et al*, 2009), we used a cutoff point of <50 for PG1 and <4 for PG1 : 2 ratio to define atrophy. There is no established cut point for PG2, and hence we used the 25th percentile (6.6 ng ml^–1^) as an arbitrary cut point. Furthermore, we analysed plasma PG1, PG2, and the PG1 : 2 ratio as quartiles and as continuous variables. For continuous analyses, consistent with our previous analyses (Kamangar *et al*, 2008; Ren *et al*, 2009), one unit change was defined as half the distance between the 25th and 75th percentiles. For PG1, PG2, and the PG1 : 2 ratio, the scaling of the continuous variables was 25, 5, and 2.5, respectively. Models were adjusted for an *a priori* group of selected variables, including additional adjustment for age beyond matching (years), plasma *H. pylori* positivity, categories of education (elementary school or less, middle school, high school, and college or higher), frequency of fruit intake (per week), frequency of vegetable intake (per week), history of ever smoking (yes *vs* no), and categories of family income (<¥10 000, ¥10 000–20 000, ¥20 000–30 000, and >¥30 000), and PG1 and PG2 were mutually adjusted.

For nonlinear continuous models we used PROG GAM with a loess smoother in SAS. Degrees of freedom were set to 3 for each model. The pepsinogen ELISA assays may not provide reliable data because of nonlinearity at high concentrations. For this reason and because the analysis is susceptible to outliers, we excluded all subjects with concentrations of PG1 >200 or PG2 >50 for this analysis (*N*=4). After fitting the model, the predicted effect size and 95% CIs were plotted on the logit scale, the scale on which a linear association would be detected. The OR comparing any two points can be calculated by subtracting the logits and exponentiating.

## Results

In all, we included 141 gastric adenocarcinoma cases and 282 controls in this analysis. Most (>90%) tumours were located in the noncardia stomach, which precluded stratifying on tumour location in any analyses. Cases and controls were matched for age, and the mean age was 58.3 years for each group (Table 1). The frequency of intake of fresh vegetables and fresh fruits, and the percentage of ever smokers, the highest educational attainment, and family income were all similar in the control and case groups.

The medians (interquartile range) of PG1, PG2, and the PG1 : 2 ratio among controls were 92.5 ng ml^–1^ (68.1–120.3), 11.4 ng ml^–1^ (6.6–16.9), and 8.7 (6.4–11.4), respectively. Of the 282 controls, 260 (92%) were positive for *H. pylori*. Of these, 251 tested positive for CagA strains whereas 9 tested negative. Because only a small number of subjects tested negative for CagA, we used any *H. pylori* positivity for further analyses. Plasma PG1 and PG2 were strongly positively correlated, with a Spearman's correlation coefficient of 0.69. In the 22 *H. pylori-*negative control subjects, the medians (interquartile range) of PG1, PG2, and the PG1 : 2 ratio were 77.6 ng ml^–1^ (57.7–92.4), 5.8 ng ml^–1^ (4.9–7.0), and 12.5 (10.3–16.0), respectively. In 260 *H. pylori-*positive control subjects, the medians (interquartile range) of PG1, PG2, and the PG1 : 2 ratio were 95.4 ng ml^–1^ (70.3–122.7), 11.9 ng ml^–1^ (7.7–17.5), and 8.4 (6.2–10.9), respectively. The differences between *H. pylori-*negative and *H. pylori-*positive subjects were significant by Wilcoxon test for each of the three measures (*P*<0.01).

In all, 92% of controls and 96.5% of cases tested positive for *H. pylori* (Table 2). Carriage of *H. pylori* more than doubled the gastric cancer risk (crude OR 2.26; 95% CI: 0.84–6.05), but this association did not reach statistical significance. Further adjustment for potential confounders including pepsinogen concentrations did not materially alter the associations. Using CagA positivity alone (96% in cases and 89% in controls) did show a significant association (OR 2.72; 95% CI: 1.09–6.78).

**Figure 1:**
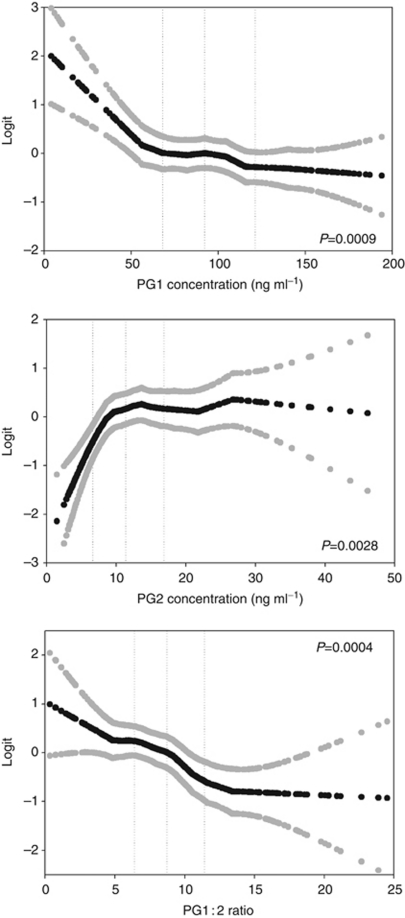
Nonlinear continuous associations between concentrations of PG1, PG2, and the PG1 : 2 ratio and odds of gastric cancer in the Shanghai Women's Health Study. The association point estimate and 95% confidence intervals between plasma concentrations and odds of gastric cancer are plotted on the logit scale as black and grey circles, respectively. Vertical dotted lines indicate the quartile boundaries for each analyte. The odds ratio for the change between any two points can be calculated by subtracting the logits and exponentiating.

Median PG1 was similar in cases and controls, but when modelled as a continuous variable we saw significantly higher risk of gastric cancer in subjects with lower PG1 concentrations (Table 2). Using a typical cutoff of <50 ng ml^–1^ we found an adjusted OR of 4.23 (95% CI: 1.86–9.63) and this value was higher than the crude estimate. Using quartiles, we saw little evidence of a trend across the distribution. In a nonlinear continuous model (Figure 1), it appears that 50 ng ml^–1^ reflects the approximate inflection point below which the risk of gastric cancer increases.

Median PG2 was similar in cases and controls and we saw borderline significantly higher risk of gastric cancer in subjects with higher concentrations of PG2 (Table 2). We estimated the risk associated with PG2 using the 25th percentile as a cutoff, and PG2 above 6.6 ng ml^–1^ was associated with an elevated risk of 3.62 (1.85–7.09) in adjusted models. Using quartiles, subjects in the upper three quartiles were at significantly higher risk than those in the first quartile. For example, subjects in the fourth quartile had an adjusted OR of 3.54 (95% CI: 1.44–8.70), but there was not a trend across quartiles; rather there appeared to be a threshold effect. In the nonlinear continuous model (Figure 1), the inflection point was ∼10 ng ml^–1^ and subjects below this level seem to have lower risk of gastric cancer.

When PG1 : 2 ratio was treated as a dichotomous variable, having a low PG1 : 2 ratio (⩽4 *vs* higher) was associated with a nonsignificantly higher risk of gastric cancer, with an adjusted OR of 1.60 (95% CI: 0.79–3.22; Table 2). When PG1 : 2 ratio was treated as quartiles, there was a significant inverse dose-response association between PG1 : 2 ratio and gastric cancer risk, with an adjusted OR of 4.54 (95% CI: 2.22–9.32) comparing the lowest *vs* highest quartiles. Likewise, there was a dose-response relationship between a continuous PG1 : 2 ratio and risk, with an adjusted OR of 1.34 (95% CI: 1.15–1.57) for each 2.50-unit decrease in PG1 : 2 ratio. The nonlinear continuous model (Figure 1) suggests a consistently decreasing risk across the PG1 : 2 ratio distribution.

To further explore the effects of having either PG1 <50 ng ml^–1^, PG2 >6.6 ng ml^–1^, or both, we fit a joint effects model (Table 3). Using those with the most favourable profile (PG1 >50 ng ml^–1^ and PG2 <6.6 ng ml^–1^) as the referent group, we found that either PG1 <50 ng ml^–1^ or PG2 >6.6 ng ml^–1^ was associated with a significantly elevated risk of approximately four-fold. When both adverse conditions were met, the adjusted odds ratio was 15.23 (95% CI: 4.49–51.63). Comparing only the highest risk group to all others produces an OR of 5.17 (95% CI: 2.01–13.31). The estimates should be interpreted cautiously because they may be overfit based on our previous knowledge of the association pattern in these four groups.

Table 4 presents gastric cancer risk in relation to PG1, PG2, and the PG1 : 2 ratio using single cut points during the cumulative 1–5 years of initial follow-up. Although case numbers were small in the first year of follow-up, and thus the precision of the estimates is low, it appears that the magnitude of the association with PG1 was strongest during the first year of follow-up with the OR of 5.18 (95% CI: 0.49–54.76). No clear pattern over time was evident for PG2. For PG1 : 2 ratio, the magnitude of the risk estimate was greatest in the first year of follow-up with the OR of 3.12 (95% CI: 0.47–20.79) and gradually reduces to the overall level with 5 years of follow-up.

## Discussion

Gastric cancer is the second most common cause of cancer death in the world (Parkin *et al*, 2005; Kamangar *et al*, 2006b). Finding methods for early detection of gastric cancer are particularly important because of its high incidence and low survival rates (Cunningham *et al*, 2005). In some countries with very high risk of gastric cancer, such as Japan, early detection programmes use upper gastrointestinal endoscopies to search for early cancerous lesions (Leung *et al*, 2008; Sugano, 2008). However, performing endoscopies in the entire population is not always feasible (Leung *et al*, 2008). Therefore, use of biological markers such as circulating pepsinogens, which indicate atrophic gastritis and the presence of other early lesions, have been used as a method to triage people who need endoscopies (Leung *et al*, 2008).

In severe atrophic gastritis, the antral-type mucosa (Kimura, 1972), which secretes only PG2, replaces fundic mucosa, which secretes both PG1 and PG2. *H. pylori-*induced inflammation of the foveolar compartment may also result in higher PG2. As a result, in subjects with severe atrophic gastritis, circulating PG1 levels are reduced substantially but circulating PG2 levels remain relatively constant (Samloff *et al*, 1982). Therefore, both PG1 and the PG1 : 2 ratio decrease (Samloff *et al*, 1982; Ley *et al*, 2001) and are associated with an elevated risk of gastric cancer (Stemmermann *et al*, 1987; Parsonnet *et al*, 1993; You *et al*, 1993; Fukuda *et al*, 1995; Ye *et al*, 2004; Kokkola *et al*, 2005; Nomura *et al*, 2005; Shiotani *et al*, 2005; Watabe *et al*, 2005; Oishi *et al*, 2006). Several studies have suggested that PG1 : 2 ratio is a more accurate marker for both atrophic gastritis (Samloff *et al*, 1982) and future risk of cancer (Stemmermann *et al*, 1987; You *et al*, 1993; Fukuda *et al*, 1995) than PG1 alone. Our results show that *H. pylori* positivity is associated with increased concentrations of both PG1 and PG2. The increase, however, is greater for PG2 than PG1 so that *H. pylori* is associated with a decreased PG1 : 2 ratio. A consistent pattern appears to be emerging, as two other recent large studies (Weck *et al*, 2007; Ren *et al*, 2009) also reported an association of *H. pylori* with increased circulating PG1 and PG2 but decreased PG1 : 2 ratio.

We examined PG1 and PG2 independently and jointly for their associations with gastric cancer risk. We found evidence of threshold effects for each of the pepsinogen concentrations such that an elevated risk was associated with PG1 <50 ng ml^–1^ or PG2 ⩾6.6 ng ml^–1^. These two phenomena appear to be independent because using each in a single risk model produced significant associations with gastric cancer risk (adjusted estimates in Table 2). When PG1 and PG2 were examined as a ratio, there is a nearly linear association between PG1 : 2 ratio and gastric cancer risk. Our results show that using either PG1 : 2 ratio quartiles Q1 *vs* Q4 (OR 4.54; 95% CI: 2.22–9.32) or the two cut points of PG1 <50 and PG2 >6.6 (OR 5.17; 95% CI: 2.01–13.31) is more strongly associated with gastric cancer risk than PG1 : 2 ratio <4 (OR 1.60; 95% CI: 0.79–3.22).

In unadjusted analyses, *H. pylori* increased gastric cancer risk by over two-fold but this association was not statistically significant. *H. pylori* is an established cause of gastric cancer, but some studies from China have shown a lower relative risk of gastric cancer associated with this bacterium compared with results reported from Western countries (Yuan *et al*, 1999; Kamangar *et al*, 2006a). The direction and magnitude of the association we observed is consistent with previous studies from China (Yuan *et al*, 1999). The lack of significance is perhaps because few people were negative for *H. pylori* and consequently there was low power to detect an association. When we used CagA positivity alone to define this exposure, a lower percentage of controls tested positive and the association became significant. This phenomenon is similar to other highly infected, high gastric cancer risk populations.

This study has several strengths that include its prospective design, collection of data on potential confounders, and rigorous methods for ascertaining outcomes. There are also some limitations. Most importantly, the sample size was modest that limited our ability to analyse data by subsites of gastric cancer and subtypes of *H. pylori*. Nevertheless, our numbers are larger than many of the recently published prospective studies of gastric cancer. Furthermore, here we only studied women and these results may not be directly applicable to men, but as noted above our results are similar to previous publications that studied both men and women.

In summary, the results of this study confirm previous findings that lower serum or plasma PG1 : 2 ratios indicate higher risk of gastric cancer and suggest that using this marker as a continuous variable is more informative of cancer risk than using it as a dichotomous variable. To our knowledge, our results show for the first time that using PG1 and PG2 as separate markers may show stronger associations with gastric cancer, but this finding needs to be replicated in other studies.

## Figures and Tables

**Table 1:** The distribution of potential confounders among gastric cancer cases and controls nested in the Shanghai Women's Health Study

	**Cases**	**Controls**	*P*-values*
*N*	141	282	—
Age, years, mean (s.d.)	58.3 (8.6)	58.3 (8.5)	—
Fresh vegetable, servings/week, mean (s.d.)	13.8 (2.5)	13.8 (2.4)	0.46
Fresh fruits, servings/week, mean (s.d.)	5.1 (3.5)	5.3 (3.2)	0.97
Ever smokers, *N* (%)	9 (6.4)	13 (4.6)	0.49
			
*Education,* N *(%)*			0.28
Elementary school or less	68 (48.2)	113 (40.1)	
Middle school	39 (27.7)	83 (29.1)	
High school	26 (18.4)	59 (20.9)	
College or higher	8 (5.7)	28 (9.9)	
			
*Family income, ¥,* N *(%)*			0.84
<10 000	29 (20.6)	50 (17.7)	
10 000 to <20 000	55 (39.0)	111 (39.4)	
20 000 to <30 000	41 (29.1)	82 (29.1)	
>30 000	16 (11.4)	39 (13.8)	

^*^P-values come from *t*-tests for continuous variables and *χ*^2^ tests for categorical variables. Age was matched and not tested for difference.

**Table 2:** The associations between gastric cancer risk and *Helicobacter pylori* seropositivity or plasma concentrations of pepsinogen 1 (PG1), pepsinogen 2 (PG2), or PG1 : 2 ratio in the Shanghai Women's Health Study

			**OR (95% CI)^a^**	
	**Cases**	**Controls**	**Crude**	**Adjusted**
H. pylori, N *(%)*				
Negative	5 (3.6)	22 (7.8)	Ref	Ref
Positive	136 (96.5)	260 (92.2)	2.26 (0.84–6.05)	2.19 (0.80–5.96)
				
*CagA,* N*(%)*				
Negative	6 (4.3)	31 (11.0)	Ref	Ref
Positive	135 (95.7)	251 (89.0)	2.77 (1.13–6.81)	2.72 (1.09–6.78)
				
*PG1 (ng ml* ^*–1*^ *)*				
Median (interquartile range (IQR))	93.0 (69.0–114.0)	92.5 (68.1–120.3)	1.22 (1.02–1.45)^b^	1.22 (1.02–1.26)^b^
⩾50, *N* (%)	120 (85.1)	263 (93.3)	Ref	Ref
<50, *N* (%)	21 (14.9)	19 (6.7)	2.65 (1.31–5.38)	4.23 (1.86–9.63)
Quartile 4 (⩾121)	29 (20.6)	70 (24.8)	Ref	Ref
Quartile 3 (93–120)	42 (29.8)	71 (25.2)	1.42 (0.82–2.48)	2.00 (1.01–3.97)
Quartile 2 (69–92)	35 (24.8)	70 (24.8)	1.19 (0.66–2.13)	1.71 (0.83–3.55)
Quartile 1 (<68)	35 (24.8)	71 (25.2)	1.20 (0.67–2.15)	1.88 (0.86–4.09)
				
*PG2 (ng ml* ^*–1*^ *)*				
Median (IQR)	12.4 (8.7–16.1)	11.4 (6.6–16.9)	1.21 (1.01–1.46)^b^	1.20 (1.00–1.44)^b^
<6.6, *N* (%)	73 (25.9)	14 (9.9)	Ref	Ref
⩾6.6, *N* (%)	209 (74.1)	127 (90.1)	3.04 (1.65–5.63)	3.62 (1.85–7.09)
Quartile 1 (<6.6)	13 (9.22)	70 (24.8)	Ref	Ref
Quartile 2 (6.6–11.3)	51 (36.2)	71 (25.2)	3.65 (1.84–7.24)	3.76 (1.87–7.59)
Quartile 3 (11.4–16.8)	46 (32.6)	70 (24.8)	3.42 (1.67–7.00)	3.98 (1.84–8.61)
Quartile 4 (⩾16.9)	31 (22.0)	71 (25.2)	2.35 (1.12–4.92)	3.54 (1.44–8.70)
				
*PG1 : 2 ratio*				
Median (IQR)	7.2 (5.6–9.2)	8.7 (6.4–11.4)	1.37 (1.18–1.59)^b^	1.34 (1.15–1.57)^b^
⩾4, *N* (%)	126 (89.4)	264 (94)	Ref	Ref
<4, *N* (%)	15 (10.6)	18 (6.4)	1.67 (0.84–3.31)	1.60 (0.79–3.22)
Quartile 4 (⩾11.4)	13 (9.2)	71 (25.2)	Ref	Ref
Quartile 3 (8.7–11.4)	29 (20.6)	70 (24.8)	2.18 (1.05–4.52)	2.08 (1.00–4.34)
Quartile 2 (6.4–8.7)	40 (28.4)	71 (25.2)	3.14 (1.52–6.48)	2.89 (1.37–6.08)
Quartile 1 (<6.4)	59 (41.8)	70 (24.8)	4.87 (2.41–9.84)	4.54 (2.22–9.32)

a Odds ratios (ORs) and 95% confidence intervals (CIs) come from conditional logistic regression models without or with further adjustment for continuous age, continuous fruit and vegetable intake, ever smoking, category of education, category of family income, and *H. pylori* seropositivity, and PG1 and PG2 were mutually adjusted.

b Continuous estimates are scaled to the average size of the two central quartiles. Specifically, the scales were for changes in concentration of −25 for PG1, 5 for PG2, and −2.5 for the PG1 : 2 ratio.

**Table 3:** The associations between gastric cancer risk and plasma concentrations of pepsinogen 1 (PG1) and pepsinogen 2 (PG2) in the Shanghai Women's Health Study using single cut points

	**Cases**	**Controls**	**OR (95% CI)^a^**	
	***N (%)***	***N (%)***	***Crude***	***Adjusted***
PG1 >50 and PG2 <6.6 ng ml^–1^	8 (6)	61 (22)	Ref	Ref
PG1 <50 and PG2 <6.6 ng ml^–1^	6 (4)	12 (4)	4.56 (1.25–16.58)	4.38 (1.17–16.36)
PG1 >50 and PG2 >6.6 ng ml^–1^	112 (79)	202 (72)	4.01 (1.86–8.66)	3.67 (1.68–8.01)
PG1 <50 and PG2 >6.6 ng ml^–1^	15 (11)	7 (2)	16.30 (4.90–54.24)	15.23 (4.49–51.63)

a Odds ratios (ORs) and 95% confidence intervals (CIs) come from conditional logistic regression models without or with further adjustment for continuous age, continuous fruit and vegetable intake, ever smoking, category of education, category of family income, *Helicobacter pylori* seropositivity, and where appropriate pepsinogens 1 and 2 concentration.

**Table 4:** The associations between pepsinogen 1 (PG1), pepsinogen 2 (PG2), and the PG1 : 2 ratio and gastric cancer risk in the Shanghai Women's Health Study by follow-up time

		**OR (95% CI)^a^**		
**Years of follow-up**	**Cases**	**PG1 <50**	**PG2 >6.6**	**PG1 : 2 ratio <4**
⩽1	16	5.18 (0.49–54.76)	2.40 (0.44–13.08)	3.12 (0.47–20.79)
⩽2	28	4.50 (0.81–25.05)	3.84 (0.90–16.35)	2.92 (0.59–14.38)
⩽3	48	2.74 (0.82–9.19)	1.73 (0.68–4.39)	2.44 (0.70–8.47)
⩽4	75	3.29 (1.17–9.27)	2.69 (1.24–5.83)	2.96 (1.05–8.35)
⩽5	90	3.95 (1.49–10.48)	3.07 (1.42–6.65)	1.76 (0.73–4.26)
				
Overall	141	4.23 (1.86–9.63)	3.62 (1.85–7.09)	1.60 (0.79–3.22)

a Odds ratios (ORs) and 95% confidence intervals (CIs) come from conditional logistic regression models with further adjustment for continuous age, continuous fruit and vegetable intake, ever smoking, category of education, category of family income, *Helicobacter pylori* seropositivity, and where appropriate pepsinogens 1 and 2 concentration.
